# Effect of Combined Cataract and Minimally Invasive Glaucoma Surgeries on Glaucoma-Specific Quality of Life

**DOI:** 10.3390/jcm15031215

**Published:** 2026-02-04

**Authors:** Jonathan T. W. Au Eong, Jin Rong Low, Eva K. Fenwick, Hla M. Htoon, Shamira A. Perera, Tina T. Wong, Ecosse L. Lamoureux, Ryan E. K. Man

**Affiliations:** 1 Singapore National Eye Centre, Singapore 168751, Singaporeshamira.perera@singhealth.com.sg (S.A.P.); tina.wong.t.l@singhealth.com.sg (T.T.W.); ryan.man@duke-nus.edu.sg (R.E.K.M.); 2 Singapore Eye Research Institute (SERI), Singapore National Eye Centre, Singapore 169856, Singapore; efenwick@duke-nus.edu.sg (E.K.F.); hla.myint.htoon@seri.com.sg (H.M.H.); 3 Duke-NUS Medical School, Singapore 169857, Singapore; 4 National University of Singapore, Singapore 117551, Singapore; 5 University of Melbourne, Melbourne, VIC 3010, Australia

**Keywords:** glaucoma, quality-of-life, minimally invasive glaucoma surgery, phacoemulsification

## Abstract

**Background**: Minimally invasive glaucoma surgery, often performed with phacoemulsification (PHACO-MIGS), for the management of primary open angle glaucoma (POAG), has good clinical outcomes and safety profiles. However, few studies have comprehensively evaluated the impact of PHACO-MIGS on patients’ quality of life (QoL). We determined the post-operative effectiveness of PHACO-MIGS on glaucoma-specific QoL domains in mild–moderate POAG patients. **Methods**: In this prospective study, adults aged ≥ 21 years with mild–moderate POAG in one eye scheduled for PHACO-MIGS at the Singapore National Eye Centre were administered a digital patient-reported outcome measure (PROM) that utilizes computerized adaptive testing (CAT) to precisely estimate glaucoma-specific QoL across 12 different domains (GlauCAT^TM^), pre-surgery and at 6 months post-surgery. The 12 domains included the following: Visual Symptoms (VSs), Ocular Comfort Symptoms (OSs), Emotional (EM), Activity Limitation (AL), Driving (DV), Lighting (LT), Mobility (MB), Treatment Convenience (TCV), Concerns (CNs), Social (SC), General Convenience (GCV), and Economic (EC). Clinical variables collected included intraocular pressure (IOP), better eye visual acuity (VA), visual field deficit (VFD) and number of glaucoma drops prescribed. Linear mixed models were utilized to determine the within-group changes in each domain, adjusted for relevant clinical, treatment and sociodemographic variables. **Results**: Of the 83 patients (mean age ± SD: 70.84 ± 6.70 years; 65.1% male; 90.4% Chinese), 61 (73.5%) underwent PHACO-MIGS with Hydrus^®^ Microstent, and 22 (26.5%) with iStent^®^ *inject*. Mean (SD) improvements in VA and IOP were observed post-surgery (0.11 [0.15] LogMAR units and 1.35 [4.20] mmHg, respectively), while VFD and the average number of anti-glaucoma medications prescribed decreased by 0.90 (2.97) dB and 1.30 (0.11) drops (all *p* < 0.05). Compared to pre-operative scores, four GlauCAT^TM^ domains [VSs (13.04%, *p* < 0.001; ES: 0.84), OSs (6.42%, *p* < 0.001; ES: 0.52), CNs (7.53%, *p* = 0.002; ES: 0.51), and GCV (6.34%, *p* = 0.004; ES: 0.45)] showed significant improvements post-surgery. The improvements across these four domains were driven primarily by a reduction in IOP and improvements in VA. **Conclusions**: Using a novel and AI-driven QoL PROM, we found significant post-operative improvements in Visual and Ocular Comfort Symptoms, Convenience, and Concerns in patients with POAG undergoing combined PHACO-MIGS, driven by improvements in IOP and VA post-surgery.

## 1. Introduction

Primary open angle glaucoma (POAG) is a progressive optic neuropathy with characteristic optic nerve and nerve fibre layer damage in the presence of an open anterior chamber angle, resulting in progressive vision loss. Indeed, this disease is the leading cause of irreversible blindness globally, with a projected increase in the number of afflicted individuals aged 40–80 years to 111.8 million in 2040 from 64.3 million in 2013, consequent to the rapidly ageing global population [1]. POAG and its associated vision loss can have a substantial impact on patients’ quality of life (QoL) [2,3,4,5].

Because POAG is incurable, timely diagnosis and life-long medication therapy using eye drops to control intraocular pressure (IOP) is central to its management paradigm [6,7]. However, patients also experience many QoL issues related with the use of glaucoma eye drops due to adverse side-effects (e.g., ocular pain and discomfort, redness), and the inconvenience of taking regular and life-long medications [8,9]. This is set to change with the introduction of minimally invasive glaucoma surgery (MIGS), an umbrella term for alternative surgical modalities for quick and efficacious reduction in IOP with minimal risks [10]. MIGS surgeries work through various mechanisms, such as bypassing the trabecular meshwork, increasing aqueous outflow through the Schlemm’s canal, increasing uveoscleral outflow, inserting an aqueous shunt through subconjunctival space, or ciliary body ablation [11]. Often performed in combination with phacoemulsification (PHACO), the combined procedure (PHACO-MIGS) has been shown to lower both IOP and reduce reliance on glaucoma medication use post-surgery, with a good safety profile [12,13,14]. PHACO-MIGS has also been shown to have a significant positive impact on QoL. For instance, a 24-month randomized controlled trial (RCT) of 505 patients comparing PHACO with PHACO-iStent^®^ *inject* found significant improvements in the driving, ocular pain and general vision subscales of the 25-item National Eye Institute Visual Function Questionnaire (NEIVFQ-25) [15]. The NEIVFQ-25, however, quantifies the QoL impact of vision impairment, which generally occurs late in the disease. As such, the NEIVFQ-25 may not reflect the true extent of QoL changes experienced by glaucoma patients post-MIGS, particularly that relating to glaucoma-specific treatment and emotional well-being.

Our group at the Singapore Eye Research Institute has developed and validated an adaptive patient-reported outcome measure (e-PROM) that utilizes computerized adaptive testing (CAT) that can precisely estimate glaucoma-specific QoL across 12 different domains, using only ~10 items per domain (GlauCAT^TM^) [16]. In this study, we report on the impact of PHACO-MIGS on glaucoma-specific QoL in mild–moderate POAG patients followed up over a six-month period using the GlauCAT^TM^. We hypothesize that, at 6 months post-PHACO-MIGS, there will be significant improvements in most of the GlauCAT^TM^ QoL domains, especially those associated with visual functioning, ocular symptoms and emotional well-being.

## 2. Methods

### 2.1. Study Population

We conducted a prospective study on patients who were scheduled for PHACO-MIGS using either Hydrus^®^ Microstent or iStent^®^ *inject* for cataract and mild-to-moderate POAG in one eye at the Singapore National Eye Centre. The study was approved by the SingHealth Institutional Review Board (CIRB Ref No: 2019/2404). All subjects provided written consent in accordance with the principles outlined in the Declaration of Helsinki prior to enrolment in this study.

Data were collected from October 2020 to December 2023. Adults aged ≥21 years scheduled for PHACO-MIGS within 3 months were recruited for the study. First contact with study participants was made by the attending physician in a specialist outpatient setting, where details of the study were explained. Informed consent was taken by another study member not involved in the care of the patient.

### 2.2. Assessment of Glaucoma-Specific QoL

The GlauCAT^TM^ is an adaptive (AI-driven) PROM designed to measure the impact of glaucoma, associated vision loss and related treatments on 12 QoL domains: Visual Symptoms (VSs), Ocular Comfort Symptoms (OSs), Emotional (EM), Activity Limitation (AL), Driving (DV), Lighting (LT), Mobility (MB), Treatment Convenience (TCV), Concerns (CNs), Social (SC), General Convenience (GCV), and Economic (EC) [16]. The QoL domains are functionally independent, and the instrument employs CAT, a “smart” algorithm that customizes the administration of questions, or “items”, to align with the respondent’s level of QoL [17]. This is achieved by selecting items for administration based on patients’ responses to preceding items. The GlauCAT^TM^ instrument was administered by trained interviewers using internet-enabled tablets during the patients’ regular clinic visits while the patients were waiting to see the attending physician at two different time points, pre-surgery and six months post-PHACO-MIGS. The scores were converted to percentiles (range 1–99 for each QoL domain; higher scores indicated better QoL outcomes) and data were stored in a secure cloud server in AWS Singapore.

### 2.3. Collection of Covariables

Baseline sociodemographic characteristics of the participants were collected via an in-house questionnaire and included age, gender and ethnicity. Presenting visual acuity (VA) of the better-seeing eye pre- and post-surgery was assessed using a logarithm of the minimum angle of resolution (LogMAR) chart at 4 m under standard lighting conditions (85 cd/m^2^). IOP (mmHg) was measured using Goldmann applanation tonometry at both baseline and follow-up, while visual field deficits (VFDs; in decibels [dB]) was assessed pre- and post-surgery using a Humphrey Field Analyser (Carl Zeiss Meditec, Jena, Germany). Anti-glaucoma topical medication prescribed were collected from participant case notes and included prostaglandin analogues, beta-blockers, alpha-adrenergic agonists, and carbonic anhydrase inhibitors.

### 2.4. Statistical Analysis

Statistical analysis was performed using STATA 17.0 (Statacorp, College Station, TX, USA). A paired samples *t*-test was used to compare the pre- and post-operative presenting VA in the better-seeing eye, number of topical anti-glaucoma medication used, IOP and VFD mean deviation. Linear mixed models were utilized to determine the within-group changes in each of the 12 GlauCAT^TM^ QoL domains, adjusted for changes in VA, IOP and VFD post-surgery, age, gender, and MIGS implant type with a random intercept for repeated measures between individuals for pre- and post-surgery measurements. A *p*-value of less than 0.05 was considered statistically significant. Effect sizes (ESs) within each QoL domain that registered statistically significant change were calculated to estimate the strength of the treatment effect (small: <0.3; medium: 0.3 to <0.5; large: ≥0.5). Dominance analysis was used to explore the relative contribution of changes in IOP, VFD, better eye presenting VA and number of topical medications to GlauCAT^TM^ domains that registered statistically significant changes post-PHACO-MIGS.

## 3. Results

### 3.1. Baseline Characteristics

A total of 83 participants (mean age ± SD: 70.84 ± 6.70 years; 65.1% male; 90.4% Chinese) were recruited (Table 1). Sixty-one patients (73.5%) underwent PHACO-MIGS with Hydrus^®^ Microstent, while 22 patients (26.5%) underwent PHACO-MIGS with iStent^®^ *inject*. Participants had a mean (SD) baseline IOP and VFD of 15.3 (4.1) and −8.52 (5.52) dB, respectively. All participants were on anti-glaucoma drops, with an average of 1.66 (range 1–4) prescribed per participant.

### 3.2. Post-Surgery Clinical Outcomes

There were significant improvements in presenting VA in the better-seeing eye and IOP in the operated eye 6 months post-surgery. The mean (SD) absolute improvements in presenting VA and IOP were 0.11 (0.15) LogMAR units and 1.35 (4.20) mmHg, respectively, while VFD decreased by −0.90 (2.97) dB (all *p* < 0.05; Table 2). Additionally, the mean number of anti-glaucoma drops prescribed decreased by an average of 1.30 (0.11) post-surgery (*p* < 0.001; Table 2), with 62 (72.9%) remaining drop-free at 6 months post-surgery.

### 3.3. Post-Surgery GlauCAT Outcomes

Compared to pre-operative scores, four GlauCAT^TM^ domains showed significant improvements post-surgery, with ESs ranging from medium to large (Table 2). These domains were the following: VSs (13.04%, *p* < 0.001; ES: 0.84), OSs (6.42%, *p* < 0.001; ES: 0.52), CNs (7.53%, *p* = 0.002; ES: 0.51), and GCV (6.34%, *p* = 0.004; ES: 0.45; Table 2).

### 3.4. Contribution of Clinical Parameters to Significant Changes in Post-PHACO-MIGS Changes in GlauCATTM Scores

For improvements in the VS domain, improvements in better eye presenting VA were the most significant contributor (38.31%) followed by IOP reduction (30.74%), VFD worsening (21.21%) and a reduction in the number of anti-glaucoma drops (9.74%). Changes in the OS domain was, however, driven primarily by IOP reduction (74.95%) and a reduction in number of anti-glaucoma drops (21.99%). Conversely, the biggest contributors to improvements in the CN and GCV domains were IOP reduction and improvement in better eye presenting VA (Table 3). Amongst the four clinical parameters evaluated, IOP contributed most to the improvements observed across the 4 GlauCAT^TM^ domains, with the % contribution ranging between 30.74% for the VS domain to 74.95% for the OS domain (Table 3).

## 4. Discussion

In our clinical cohort study evaluating post-surgical changes in 12 different glaucoma-specific QoL domains measured using the GlauCAT^TM^ after combined PHACO-MIGS in patients with POAG, we found statistically significant improvements in VSs, OSs, CNs and GCV. Absolute improvements were also noted in six of the remaining eight GlauCAT^TM^ domains, although these did not reach statistical significance. IOP reduction was the clinical parameter contributing most to the significant improvements observed across the four GlauCAT^TM^ domains. Our findings suggest that the PHACO-MIGS results in substantial improvements in glaucoma-specific QoL domains related to Visual and Ocular Comfort Symptoms, Convenience, and Concerns, with this improvement driven primarily by the post-surgical improvement in IOP. The results of our study not only help to bridge the gap in the literature on the impact of PHACO-MIGS on patient-reported outcomes but also provides clinicians with evidence-based information with which to counsel patients on treatment-related outcomes during clinical consultations. At a systems level, it can also provide valuable information to better situate this combined surgical procedure in the current glaucoma management paradigm for mild–moderate POAG [18].

Thus far, MIGS research has been largely focused on its clinical efficacy and safety profile with only recently emerging studies addressing patient-reported outcomes. These studies have reported improvements in patients’ QoL post-PHACO-MIGS, though these studies have largely focused on vision-related and generic QoL using PROMs such as the NEIVFQ-25, Ocular Surface Disease Index (OSDI), and EuroQoL 5-Dimensions scale (EQ-5D) [15,19,20,21]. Our improvements in the VS, OS, GCV and CN GlauCAT^TM^ QoL domains are likely due to improvements in VA secondary to cataract extraction [22,23] and a significant reduction in side effects associated with a post-operative decreased reliance on anti-glaucoma medication use [19,24], as supported by our dominance analysis. Somewhat unexpectedly, we observed that a reduction in IOP was also a key driver of the observed improvements in all four domains. The prevailing consensus is that a reduction in IOP of the observed magnitude is unlikely to result in symptomatic changes, particularly where the baseline IOP is not high enough to be symptomatic (e.g., pain, visual blurriness due to corneal edema) [25,26]. The substantial contribution of IOP reduction to post-surgical improvements in GlauCAT^TM^ scores may be attributed to its role as a surrogate marker for surgical success, which subsequently leads to a reduction in medication use [27,28]. Indeed, we observed that, when post-surgical change in IOP was excluded, the decrease in the number of prescribed glaucoma medications emerged as the primary contributor (94.89% of the observed variance) to improvements in the OS domain, supporting the hypothesis. Further research involving larger sample sizes is warranted to better understand the potential mediating effect of reduced IOP on the observed improvements in glaucoma-specific QoL post-PHACO-MIGS.

Additionally, we did not observe any improvements in the TCV domain; however, this is likely because all but two of the items in this domain relate to QoL challenges specific to anti-glaucoma eye drops which, after PHACO-MIGS, were not applicable to patients. This meant that improved convenience related to ceasing anti-glaucoma eye drops, although likely to be considerable, was not captured with the current version of the TCV domain, which is unfortunate, as achieving a state free from topical medication is considered a key clinical outcome for the PHACO-MIGS procedure. To overcome this issue, future work will aim to modify the content of the TCV items to capture such treatment-related changes. Our study was also not able to elicit improvements to the EC domain post-PHACO-MIGS, similar to the study reported by Al-Habash and colleagues, which found no change in the self-reported economic status of their study participants post-surgery [21]. Aside from the fact that these results are highly healthcare system-dependent, the observed outcome may be attributed to the initial expense associated with the PHACO-MIGS procedure, which potentially offsets the cost savings achieved through the decreased need over time for anti-glaucoma medications following surgery. Larger studies focusing on eliciting the cost-effectiveness of the PHACO-MIGS procedure over a longer time-period is warranted.

The strengths of our study include a well-described clinical sample and the use of a standardized protocol in patient recruitment and follow-up, as well as a validated glaucoma-specific PROM in the evaluation of QoL. Post-operatively, eye drops were stopped and only instituted again at the discretion of the ophthalmologist depending on the target IOP. This standardized methodology allowed singular aims like seeing how many patients were drop-free to be obtained. However, this study has some limitations that should be acknowledged. First, our study had a relatively small sample size of 83 patients, which may have affected its statistical power to detect significant differences post-surgery. Indeed, post hoc power calculations revealed that the statistical power for each of the non-significant associations between the PHACO-MIGS procedure and GlauCAT^TM^ ranged between 6.3% and 48.6%, which is below the recommended 80% statistical power to rule out false negative (Type II) errors [29]. Secondly, the limited follow-up period of six months in our study precluded the examination of long-term changes in glaucoma-specific quality of life (QoL). This limitation is significant, as the potential of the PHACO-MIGS procedure to decrease the frequency of future incisional surgeries and its consequent impact on QoL could not be determined within the constraints of our short follow-up period. Third, the GlauCAT^TM^ was developed and validated in a largely Caucasian population, which may have led to inaccurate estimates of QoL when administered to an Asian population. Fourth, we could not determine the influence of the individual procedures (PHACO alone or MIGS only) on the QoL outcomes, nor if differences in MIGS procedures would affect these outcomes, due to an absence of a PHACO-alone group and a lack of statistical power to conduct stratified analyses, respectively. Lastly, we did not assess health-state (utility) preferences for these patients, which are critical to cost–utility analyses. A larger study is underway to evaluate the glaucoma-specific QoL and utility changes post-PHACO-MIGS, relative to both PHACO alone and continued medication use only, using glaucoma-specific QoL and utility instruments developed and validated in Asian populations [30,31].

## 5. Conclusions

In summary, using a novel, adaptive, glaucoma-specific QoL PROM, we found statistically significant improvements in Visual and Ocular Comfort Symptoms, Convenience, and Concerns, in patients with POAG undergoing combined PHACO-MIGS in a tertiary eye institute in Singapore. With the shift towards value-based healthcare within the Singaporean clinical care system [20], our study provides timely evidence to support improved glaucoma-specific QoL post-PHACO-MIGS, which can help inform clinical management guidelines for patients with mild–moderate POAG. Nonetheless, larger studies are warranted to determine the patient-reported outcomes and cost-effectiveness of this combined procedure as compared to PHACO-only or PHACO-trabeculectomy, as well as continued medication use only, which remains the gold standard first-line treatment for POAG.

## Figures and Tables

**Table 1:** Baseline sociodemographic and clinical characteristics of the 83 study participants.

Characteristics		N (%) or Mean ± SD
Age (years)		70.84 ± 6.70
Race	Chinese	75 (90.4)
	Malay	4 (4.8)
	Indian	4 (4.8)
Gender	Male	54 (65.1)
	Female	29 (34.9)
Baseline IOP (mmHg)		15.29 ± 4.13
Number of glaucoma medications prescribed pre-surgery		1.66 (0.75)/range 1–4
Visual field deficit (dB)		−8.52 ± 5.52
Presenting VA of the better-seeing eye (LogMAR)		0.23 ± 0.14

dB = decibels; IOP = intraocular pressure; LogMAR = logarithm of the minimum angle of resolution; SD = standard deviation; dB = decibels.

**Table 2:** Comparison of surgical outcomes and GlauCAT^TM^ quality-of-life domains after PHACO-MIGS (N = 83).

**Surgical Outcomes Post-Surgery**						
**Outcome Measure**	**Mean Difference (SD)**			***p*-Value**		
IOP (mmHg)	1.35 (4.20)			0.005		
Visual field deficit (dB)	−0.90 (2.97)			0.010		
Presenting visual acuity in better-seeing eye (LogMAR)	0.11 (0.15)			<0.001		
Number of anti-glaucoma medications prescribed	−1.30 (0.11)			<0.001		
**GlauCAT^TM^ quality-of-life domains**						
**Domains**	**Pre-surgery (mean [SD])**	**Post-surgery (mean [SD])**	**Difference**	***p*** **-value**	Effect size *	**Statistical power (%)**
Visual Symptoms	72.76 (16.59)	85.81 (14.33)	13.04	<0.001	0.842	
Ocular Comfort Symptoms	79.84 (13.05)	86.26 (11.64)	6.42	0.001	0.519	
Emotional	90.98 (14.47)	94.07 (13.40)	3.09	0.155		48.6
Activity Limitation	91.21 (12.99)	94.24 (13.13)	3.03	0.139		54.7
Driving	60.39 (19.64)	61.85 (20.74)	1.46	0.641		9.7
Lighting	91.01 (13.74)	92.24 (16.41)	1.26	0.406		10.6
Mobility	97.32 (7.58)	97.74 (7.31)	0.28	0.811		6.3
Treatment Convenience	89.79 (16.29)	88.78 (15.11)	−1.02	0.677		8.7
Concerns	77.88 (15.72)	85.52 (14.49)	7.53	0.002	0.505	
Social	96.17 (9.61)	97.09 (8.90)	0.72	0.619		10.4
General Convenience	85.64 (15.34)	91.98 (12.52)	6.34	0.004	0.453	
Economic	84.32 (22.82)	83.06 (26.27)	−1.26	0.741		7.2

SD: standard deviation, IOP: Intraocular pressure, LogMAR: logarithm of the minimum angle of resolution, dB: decibels. * Calculated for *p* < 0.05 only.

**Table 3:** Relative contribution of changes in key clinical parameters post-PHACO-MIGS to changes in GlauCATTM domains.

	% Contribution			
Clinical Parameters	Visual Symptoms	Ocular Comfort Symptoms	Concerns	Convenience-General
Intraocular pressure	30.74%	74.95%	51.20%	55.81%
Visual field deficit	21.21%	1.95%	1.71%	15.96%
Better eye presenting VA	38.31%	1.11%	42.25%	26.36%
No. of topical drops	9.74%	21.99%	4.84%	1.87%

PHACO-MIGS: combined phacoemulsification and minimally invasive glaucoma surgery; VA: visual acuity.

## Data Availability

The data presented in this study are available on request from the corresponding author.
